# The genus *Sorolopha* Lower, 1901 (Lepidoptera, Tortricidae, Olethreutinae) in China: an annotated checklist, new DNA barcodes, and descriptions of two new species

**DOI:** 10.3897/zookeys.1295.202313

**Published:** 2026-10-20

**Authors:** Zhurong Hu, Xiuping Chang, Yuxuan Ma, Ershuai Li, Changyi Guo, Haili Yu

**Affiliations:** 1 Shaanxi Key Laboratory for Animal Conservation, Northwest University, Xi’an, 710069, China Shaanxi Key Laboratory for Animal Conservation, Northwest University Xi’an China; 2 Shaanxi Key Laboratory of Qinling Ecological Security, Shaanxi Academy of Sciences, Xi’an, 710043, China Shaanxi Key Laboratory of Qinling Ecological Security, Shaanxi Academy of Sciences Xi’an China

**Keywords:** Genetic divergence, larval biology, new records, Olethreutini, *Sorolopha irirectangulata* sp. nov., *Sorolopha specialis* sp. nov.

## Abstract

This study presents a detailed checklist of the genus *Sorolopha* from China, based on examination of museum specimens, published records, and recent fieldwork. A total of 36 species are recognized and documented from China, including information on type depositories, type localities, distributions, relevant literature, and available biological data. Two species, *S. irirectangulata* Hu & Yu, **sp. nov**. and *S. specialis* Hu & Yu, **sp. nov**., are described as new to science. Four species, *S. baribaris* Sakagami, 2023, *S. bathysema* Diakonoff, 1973, *S. ghilarovi* Kuznetsov, 1988, and *S. suthepensis* Patibhakyothin & Pinkaew, 2015, are newly recorded from China, and the female gentitalia of *S. suthepensis* are described for the first time. DNA barcodes are newly reported for 15 species, together with a maximum-likelihood tree and an analysis of genetic divergence to aid species delimitation. Illustrations of adults and genitalia of the new species are provided, with images of the larva and pupa of *S. herbifera* (Meyrick, 1909). A key to the Chinese species based on male genitalia and a distribution map of *Sorolopha* in China are also included.

## Introduction

The genus *Sorolopha* Lower, 1901 is one of the most species-rich genera in the tribe Olethreutini (Lepidoptera: Tortricidae: Olethreutinae). It currently comprises 77 species distributed across the Oriental, Australasian, Palaearctic, and African regions, with the majority occurring in the Oriental region (Gilligan et al. 2018; Lopez-Vaamonde et al. 2019; Sakagami 2023). Adults of *Sorolopha* are medium-sized and generally recognizable by their distinctive appearance: most species have forewings with green shading and a prominent, rounded marking in the apical region. Males often have lateral brushes of sex scales on the abdomen, and their genitalia are characterized by extremely long, pendulous socii and slender valva. The larvae are leaf rollers, and host records indicate associations with nine plant families: Elaeocarpaceae, Fabaceae, Lauraceae, Meliaceae, Myrtaceae, Pandanaceae, Magnoliaceae, Schisandraceae and Saxifragaceae (Diakonoff 1973; Horak 2006; Brown et al. 2008, 2019; Robinson et al. 2023; Sakagami 2023). To date, 47 standard 658-bp COI fragments representing at least 11 *Sorolopha* species are available in GenBank and BOLD (accessed 1 April 2026).

Previously, 30 species of *Sorolopha* were reported from China (Meyrick 1912, 1931, 1936; Wileman and Stringer 1929; Falkovitsh 1966; Liu and Bai 1982, 1985; Kawabe et al. 1992; Liu and Li 2002; Yu and Li 2009; Zhao et al. 2017; Sun et al. 2017; Li et al. 2023; Sun and Li 2024). These species are primarily distributed in the humid regions of southern China, with the highest diversity observed in Yunnan, Guangdong, Hainan, and Taiwan Provinces. No *Sorolopha* species has yet been reported to cause significant economic damage in China. Liu and Li (2002) documented two host plants from China: *Litsea glutinosa* (Lauraceae) for *S. archimedias* (Meyrick, 1912), and *Michelia fuscata* (Magnoliaceae) for *S. camarotis* (Meyrick, 1936).

Recently, the authors examined Chinese olethreutine specimens deposited in the collections of Nankai University and Northwest University, China. Six previously unrecorded *Sorolopha* species were identified, including two new species—*S. specialis* sp. nov. and *S. irirectangulata* sp. nov.—and four species formerly unknown from China: *S. baribaris* Sakagami, 2023, *S. bathysema* Diakonoff, 1973, *S. ghilarovi* Kuznetsov, 1988, and *S. suthepensis* Patibhakyothin & Pinkaew, 2015. These additions raise the total number of *Sorolopha* species recorded from China to 36, accounting for approximately half of the genus’s total diversity. The present study aims to provide an overview of the *Sorolopha* species found in China and to report new data, including biological information for one species and 18 newly generated COI sequences (658 bp) representing 15 species.

## Materials and methods

This study is based mainly on specimens deposited in the insect collections of Northwest University (Xi’an, China) and Nankai University (Tianjin, China). The types of the new species are deposited in the Insect Collection of Nankai University, Tianjin, China (**NKU**). Specimens were collected using light traps in the field. All specimens were examined under an Olympus SZX71 stereomicroscope. Genitalia dissection followed the methods described by Li (2002). Both adults and genitalia were photographed using a digital microscope (VHX-5000).

Larvae were collected from garden plants in Guangzhou, China, and reared on their host plants in plastic cases under ambient conditions. The host plants were identified by the collector, who is a biologist. Emerged adult moths were identified based on morphological characteristics, including the genitalia. Photographs of larval leaf shelters in the field were taken using a mobile phone, while images of the larva and pupa were captured with a Canon EOS 5D camera equipped with a macro lens.

Total genomic DNA was extracted with the Genomic DNA Extraction Kit (Tiangen Biotech., Beijing, China) from legs removed from dried adult specimens according to the manufacturer’s instructions. Genomic DNA was eluted into 50 μL TE buffer and stored in a freezer (−20 °C). The target fragments of COI were amplified as described by Hebert et al. (2004). The sequences were aligned using ClustalW in MEGA 7 (Kumar et al. 2016) and the genetic distance was analyzed using the Kimura 2-Parameter (K2P) model. Maximum-likelihood (ML) analysis was performed in IQ‑TREE v. 2.3.4 (Nguyen et al. 2015) with the GTR+I+G4+F nucleotide-substitution model, based on the aligned COI dataset. Nodal support was evaluated with 1,000 replicates of the SH-aLRT (Guindon et al. 2010), 1,000 standard non-parametric bootstrap replicates (Felsenstein 1985), and the approximate Bayes test (Anisimova et al. 2011). Six outgroup sequences representing four genera were retrieved from GenBank. The outgroup set included one sequence of *Polylopha epidesma* Lower, 1901 (Chlidanotinae, the basal subfamily of Tortricidae), one sequence of *Cryptaspasma brachyptycha* (Meyrick, 1911), belonging to the most basal genus of Olethreutinae; and one sequence each of *Penthostola subnigrantis* Groenen & Wangdi, 2019, *P. tricolorana* Kuznetzov, 2000, *Eudemis porphyrana* (Hubner, [1796–1799]), and *E. profundana* (Denis & Schiffermuller, 1775), which represent genera closely related to the focal genus. The ML tree was constructed primarily for visualizing species clustering and assessing congruence with morphologically defined species boundaries, rather than for formal phylogenetic inference. The sequence data obtained in this study have been deposited in GenBank with the accession numbers PX852817–PX852832, PX852848–PX852849.

## Museum acronyms

**NHMUK** The Natural History Museum, London, United Kingdom

**IZAS** Institute of Zoology, Academia Sinica, Beijing, China

**KKIC** Kasetsart Kamphaeng Saen Insect Collection, Nakhon Pathom, Thailand

**MNHU** Museum fur Naturkunde der Humboldt-Universitat, Berlin, Germany

**MGAB** Museum Gunung Api Batur, Bangli, Indonesia

**MGCL** McGuire Center for Lepidoptera, Florida Museum of Natural History, Gainsville, USA

**NCB** Nederlands Centrum voor Biodiversiteit, Leiden, Netherlands

**NKU** Nankai University, Tianjin, China

**NMNS** National Museum of Nature and Science, Ibaragi, Japan

**NWU** Northwest University, Xi’an, China

**OMU** Osaka Metropolitan University, Sakai, Japan

**OPU** Entomological Laboratory, Osaka Prefecture University, Sakai, Japan

**SAMA** South Australian Museum, Adelaide, Australia

**ZISP** Zoological Institute, Russian Academy of Sciences, St. Petersburg, Russia

## Results

### Molecular study

Forty-seven standard 658-bp COI barcode sequences of *Sorolopha* were available in GenBank and BOLD (accessed 1 April 2026), which derived from specimens collected in Australia, Vietnam, Papua New Guinea, and Madagascar. Among these sequences, 15 had not been identified to the species level, while the remaining 32 belonged to 11 known species. In the present study, we successfully sequenced standard COI barcode fragments (658 bp) from 18 exemplar specimens representing 15 *Sorolopha* species collected in China. Combined with previously available sequences, our final dataset comprised 71 COI sequences (658 bp) spanning at least 25 *Sorolopha* species and six outgroup species (Suppl. material 1: table S1).

**Figure 1.**
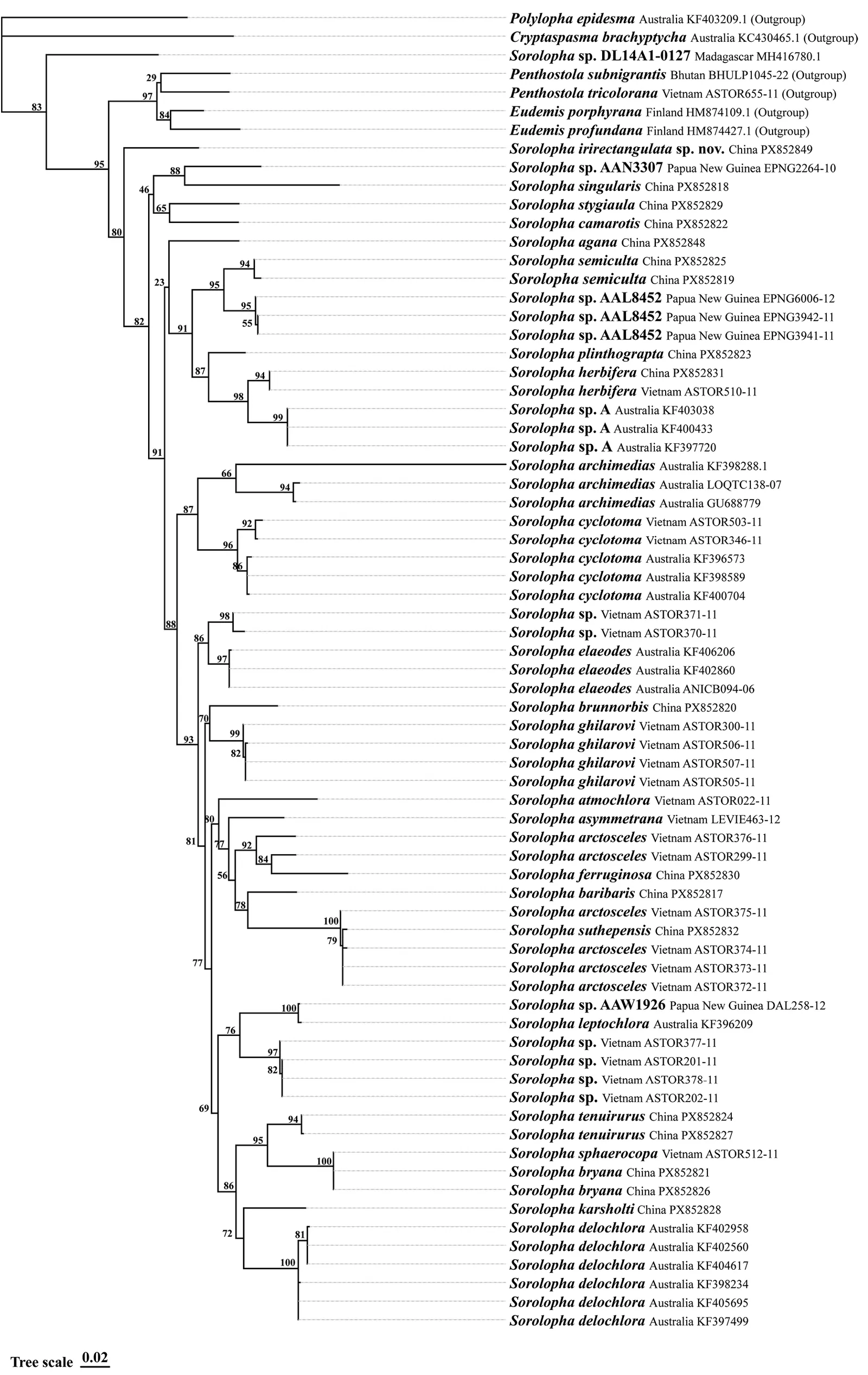
Maximum-likelihood tree based on DNA barcodes of *Sorolopha* species. Numbers above/below branches refer to bootstrap values.

A genetic distance of ~ 2% has been widely observed to correspond reasonably well with morphology-based species delimitations in many Lepidoptera (Hajibabaei et al. 2006; Zahiri et al. 2014; Lee et al. 2018). Studies focusing on Tortricidae largely support this pattern (Hulcr et al. 2007; Gilligan et al. 2016; Vargas-Ortiz et al. 2017; Corley and Ferreira 2017; Jia et al. 2025). Applying this criterion, our maximum-likelihood (ML) analysis of the 71 sequences (Fig. 1) resolved 32 putative species-level clades of *Sorolopha*, each strongly supported by high bootstrap values (99–100%). One unidentified sequence from Madagascar (GenBank accession no. MH416780.1), although included among the 65 sequences analyzed, fell outside the clade of *Sorolopha* and appeared as a highly divergent outlier in the ML tree. Because its anomalous position likely reflects a misidentification of the deposited sequence in GenBank, this sequence was excluded from the K2P genetic-distance analysis; it was, however, retained in the ML tree to ensure complete representation of the dataset.

Pairwise Kimura 2-parameter (K2P) genetic distances based on 64 COI sequences among *Sorolopha* species are summarized (Suppl. material 2: table S2: Pairwise distances calculated within and between *Sorolopha* species resulting from COI gene dataset). Intraspecific K2P distances were calculated for 15 *Sorolopha* species-level clades, each with bootstrap support ≥ 94%. The maximum intraspecific distance was 2.38%, with a mean intraspecific divergence of 0.33%. The overall mean interspecific genetic divergence was 9.00%. For all 31 species-level clades (including those represented by a single sequence), the nearest neighbor interspecific distance ranged from 2.89% to 11.80%.

### Systematic account

#### Sorolopha

Taxon classificationAnimaliaLepidopteraTortricidae

Lower, 1901

1E06409A-E2DC-5B0F-8B1C-4F2B998006E8

Sorolopha Lower, 1901: 319. Type species: Sorolopha cyclotoma Lower, 1901, by monotypy.
Acanthothyspoda Lower, 1908: 319. Type species: Acanthothyspoda elaeodes Lower, 1908, by original designation.
Alypeta Turner, 1916: 528. Type species: Alypeta delochlora Turner, 1916, by original designation.
Chogantha Razowski, 1960: 387. Type species: Argyroploce sphaerocopa Meyrick, 1930, by original designation.

##### Notes.

**Figure 2.**
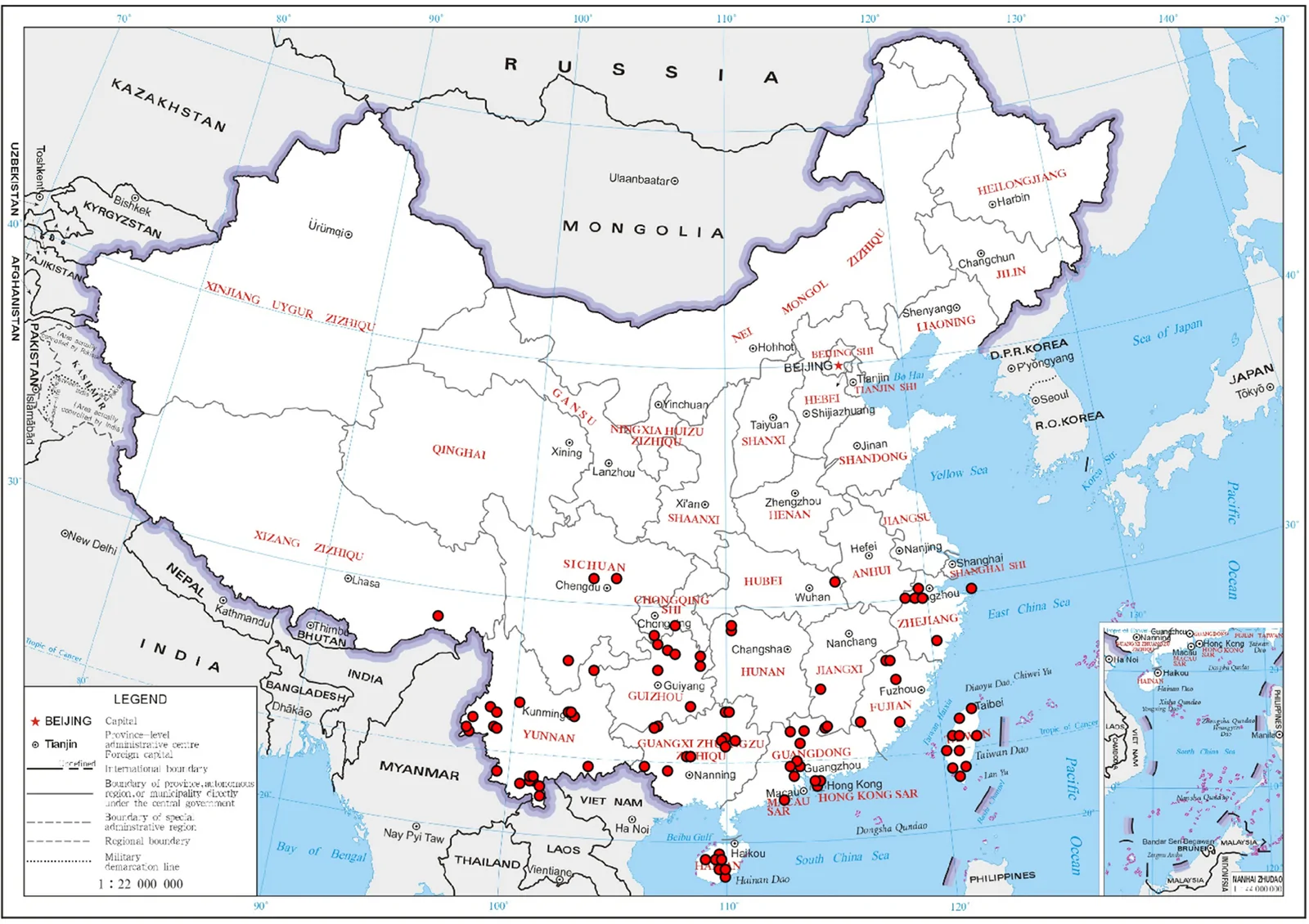
Distribution of *Sorolopha* species in China.

Species of *Sorolopha* in China are restricted to areas south of 31°N latitude and below 3,100 meters in elevation (Fig. 2), and all specimens were collected from habitats with well-developed vegetation. In this study, two species are recognized as new: *S. specialis* sp. nov. and *S. irirectangulata* sp. nov., and four species are newly recorded for China: *S. baribaris* Sakagami, 2023, *S. bathysema* Diakonoff, 1973, *S. ghilarovi* Kuznetsov, 1988, and *S. suthepensis* Patibhakyothin & Pinkaew, 2015. Also, the female of *S. suthepensis* is reported for the first time.

### Key to Chinese species of *Sorolopha* based on male genitalia

**Table:** 

1	Valva with a digitate costal prominence near base	***S. dactyloidea***
–	Valva lacking a costal prominence	**2**
2	Socius ~ 2/3 the length of tegumen	**3**
–	Socius < 1/2 the length of tegumen	**8**
3	Sacculus without long spines	**4**
–	Sacculus with a cluster of long spines on ventral edge	**6**
4	Tegumen longer than cucullus; cucullus with basal 2/3 densely setose, apex sparsely setose, median part as wide as apical part; ventral prominence rounded; base of phallus narrower than middle of cucullus	***S. bathysema***
–	Tegumen equal to cucullus in length; cucullus sparsely and uniformly setose throughout, except for dense spines along basal 1/4 of ventral edge, median part narrower than apical part; ventral prominence distally straight and broad; base of phallus wider than middle of cucullus	**5**
5	Sacculus with a few distal spines along outer edge of basal excavation; basal prominence of cucullus slightly separated from sacculus; phallus ~ 2/3 the length of socius	***S. agana***
–	Sacculus with dense distal spines extending from outer edge of basal excavation to ventral edge of sacculus; basal prominence of cucullus adjacent to sacculus; phallus < 1/2 the length of socius	***S. saitoi***
6	Valva with a distinct, naked neck; costa with two long, stout thorns at 1/3 length	***S. stygiaula***
–	Valva without a neck; basal half of costa nearly naked, without thorns	**7**
7	Gnathos naked medially; sacculus with two separate bristle clusters; ventral prominence of cucullus distinct and triangular, bearing four tooth-like apical thorns	***S. camarotis***
–	Gnathos aciculate medially; sacculus with two adjacent bristle clusters; ventral prominence of cucullus indistinct, bearing a single strong apical thorn	***S. singularis***
8	Valva without a neck, cucullus slightly expanded medially	***S. archimedias***
–	Valva with a distinct neck, cucullus expanded medially or distally, forming a distinct ventral prominence	**9**
9	Valvae asymmetrical	**10**
–	Valvae symmetrical	**19**
10	Gnathos strongly sclerotized and medially broadened; left valva with a small, naked triangular lobe on neck	***S. ghilarovi***
–	Gnathos membranous or weakly sclerotized, not broadened medially; valvae without lobes on neck	**11**
11	Ventral prominences of left and right cucullus differing in shape, bearing several long thorns	**12**
–	Ventral prominences of left and right cucullus identical in shape, without long thorns	**13**
12	Ventral prominence of cucullus club-shaped, longer than apical part of cucullus	***S. bryana***
–	Ventral prominence of cucullus trapezoidal, shorter than apical part of cucullus	***S. tenuirurus***
13	Left and right sacculus roughly with same width, both narrow spindle-shaped	**14**
–	Left sacculus obtusely square, wider than right sacculus, right sacculus narrow spindle-shaped	**17**
14	Ventral prominence of cucullus longer than wide	***S. aeolochlora***
–	Ventral prominence of cucullus wider than long	**15**
15	Valva with neck constricted apically and bent ventrally	***S. karsholti***
–	Valva with neck not constricted apically, straight	**16**
16	Left cucullus with apical part longer than ventral prominence	***S. muscida***
–	Left cucullus with apical part equal in length to ventral prominence	***S. identaeolochloca***
17	Left sacculus with ventral edge less angled; right sacculus without short setae towards neck	***S. suthepensis***
–	Left sacculus with ventral edge angled; right sacculus with short setae towards neck	**18**
18	Apex of cucullus rounded	***S. asphaeropa***
–	Apex of cucullus oblong	***S. sphaerocopa***
19	Ventral prominence of cucullus square distally	***S. elaeodes***
–	Ventral prominence of cucullus rounded or triangular distally	**20**
20	Ventral prominence of cucullus rounded distally	**21**
–	Ventral prominence of cucullus triangular, pointed distally	**27**
21	Cucullus with apical part wider than ventral prominence	***S. irirectangulata* sp. nov**.
–	Cucullus with apical part narrower than ventral prominence	**22**
22	Cucullus with distal half extremely narrowed, ~ 1/2 as wide as the neck of valva	***S. cyclotoma***
–	Cucullus with distal half wider than the neck of valva	**23**
23	Valva with neck ~ 1/2 as wide as its widest part	***S. latiuscula***
–	Valva with neck distinctly narrower than 1/2 the width of its widest part	**24**
24	Cucullus shorter than 1/2 the length of valva	***S. specialis* sp. nov**.
–	Cucullus longer than 1/2 the length of valva	**25**
25	Cucullus with apex 1/2 as wide as its widest part	***S. ferruginosa***
–	Cucullus with apex 2/3 as wide as its widest part	**26**
26	Length of the basal 1/2 of valva ~ 3× its maximum width	***S. baribaris***
–	Length of the basal 1/2 of valva no more than 2.5× its maximum width	***S. nanlingica***
27	Phallus no more than 1.3× the length of socius	**28**
–	Phallus > 1.5× the length of socius	**31**
28	Sacculus with ventral edge protruding to a right angle; cucullus bearing a cluster of long spines along proximal edge of ventral prominence	***S. herbifera***
–	Sacculus with ventral edge arched; cucullus without long spine clusters along proximal edge of ventral prominence	**29**
29	Socius apically reaching gnathos; cucullus with ventral prominence located at 2/5 of ventral edge	***S. rubescens***
–	Socius apically not reaching gnathos; cucullus with ventral prominence basally	**30**
30	Uncus pointed apically; cucullus with ventral prominence bearing an apical thorn	***S. brunnorbis***
–	Uncus rounded apically; cucullus with ventral prominence without thorns apically	***S. angustorbis***
31	Neck of valva narrower than the narrowest part of cucullus	**32**
–	Neck of valva wider than or equal in width to the narrowest part of cucullus	**33**
32	Cucullus gradually narrowed from ventral prominence to apex, ventral edge beyond the prominence straight	***S. euochropa***
–	Cucullus widened apically, ventral edge beyond the ventral prominence concave	***S. phyllochlora***
33 Phallus more than 1.7× the length of socius	**34**
–	Phallus less than 1.5× the length of socius	**35**
34	Socius dilated apically, with apex > 2× basal part in width; cucullus with distal part gradually broadened to apex, basal part of ventral edge covered by sparsely short spines and lacking long spine clusters	***S. semiculta***
–	Socius with apex slightly broader than basal part; cucullus with distal part uniform in width, basal part of ventral edge covered by dense short spines and a series of long spines	***S. plinthograpta***
35	Sacculus with ventral edge arched; cucullus with ventral prominence angular, not elongated apically	***S. liochlora***
–	Sacculus with ventral edge projecting at a right angle; cucullus with ventral prominence elongated apically, bird-beak-like	***S. plumboviridis***

#### Sorolopha aeolochlora

Taxon classificationAnimaliaLepidopteraTortricidae

(Meyrick, 1916)

7FBADC3F-DE8B-509F-B2EB-5543096DC899

Argyroploce aeolochlora Meyrick, 1916: 562. TL: India. TD: NHMUK.
Choganhia aeolochlora : Razowski, 1960: 387.
Eudemis aeolochlora : Diakonoff, 1968: 51.
Sorolopha aeolochlora : Diakonoff, 1973: 88.

##### Distribution.

China (Taiwan) (Kawabe et al. 1992; Sun and Li 2024), India (Meyrick 1916; Diakonoff 1973; Pathania et al. 2020; Naik and Shashank 2024), Thailand (Kawabe 1989).

##### Remarks.

Specimens from China were collected at elevations of 650–2,750 m.

#### Sorolopha agana

Taxon classificationAnimaliaLepidopteraTortricidae

(Falkovitsh, 1966)

9F3FB958-55C9-5547-AEFC-6DCA5E487780

Choganhia agana Falkovitsh, 1966: 209. TL: China. TD: MGAB.
Sorolopha agana : Diakonoff, 1973: 95.

##### Distribution.

China (Guangxi, Guizhou, Hainan, Hunan, Jiangxi, Sichuan, Taiwan, Zhejiang) (Falkovitsh 1966; Liu and Bai 1982; Liu and Li 2002; Yu and Li 2009; Sun and Li 2024).

##### Remarks.

Specimens from China were collected at elevations of 50–1,450 m.

#### Sorolopha angustorbis

Taxon classificationAnimaliaLepidopteraTortricidae

Sun, Sun & Heppner, 2017

FC8785C5-20EB-50A3-BB35-AB263742280C

Sorolopha angustorbis Sun et al., 2017: 642. TL: China. TD: MGCL.

##### Distribution.

China (Taiwan) (Sun et al. 2017).

##### Remarks.

Specimens from China were collected at elevations of 650–1,200 m.

#### Sorolopha archimedias

Taxon classificationAnimaliaLepidopteraTortricidae

(Meyrick, 1912)

0883285C-29D5-5DD4-BE89-A659E9C4F7A8

Argyroploce archimedias Meyrick, 1912: 63. TL: China. TD: NHMUK.
Olethreutes purpuissatana : Clarke, 1958: 540.
Eudemis archimedias : Falkovitch, 1962: 191.
Eudemis oxygona Diakonoff, 1968: 50.
Sorolopha archimedias : Diakonoff, 1973: 54.

##### Host plants.

Lauraceae: *Cinnamomum camphora*, *C. iners*, *C. verum*, *Litsea chinensis*, *L. glutinosa*; Myrtaceae: *Eugenia* sp., *Syzygium aromaticum* (Diakonoff 1973; Horak 2006; Brown et al. 2008; Robinson et al. 2023).

##### Distribution.

China (Guangdong, Guangxi, Hainan, Hong Kong, Hubei, Yunnan, Zhejiang) (Meyrick 1912; Yu and Li 2009; Li et al. 2023), Philippines (Diakonoff 1968), Indonesia, Sri Lanka, India (Diakonoff 1973, 1982; Naik and Shashank 2024), Australia (Horak 2006), Thailand (Pinkaew 2007), Vietnam (Le et al. 2013), Laos (Pinkaew and Phewphanh 2017).

##### Remarks.

Specimens from China were collected at elevations of 130–1,200 m.

#### Sorolopha asphaeropa

Taxon classificationAnimaliaLepidopteraTortricidae

Diakonoff, 1973

213B84CE-3339-55B4-8560-6CA8AA37EEC0

Sorolopha asphaeropa Diakonoff, 1973: 87. TL: New Guinea. TD: NHMUK.

##### Distribution.

China (Guangxi) (Yu and Li 2009), New Guinea (Diakonoff 1973).

##### Remarks.

Specimens from China were collected at an elevation of 510 m.

#### Sorolopha baribaris

Taxon classificationAnimaliaLepidopteraTortricidae

Sakagami, 2023

29ABD5AD-E6D1-58B1-B949-07E2580D6E6E

Sorolopha baribaris Sakagami, 2023: 138. TL: Japan. TD: NMNH.

##### Specimens examined.

China • 1 ♂; Zhejiang Prov., Lin’an, Mt. Tianmu; 30°19'N, 119°27'E; alt. 360 m; 26 Jun. 2013; A.H. Yin and X.C. Wang leg. • 1 ♂; Zhejiang Prov., Lishui, Mt. Jiulong; 28°23'N, 118°50'E; alt. 470 m; 6 Aug. 2013; A.H. Yin and X.C. Wang leg. • 1 ♂; same collection data as for preceding; alt. 510 m; 26 Aug. 2017; S.N. Qian, Y.Y. Jia, and J. Li leg.

##### Host plant.

Lauraceae: *Actinodaphne acuminata* (Sakagami 2023).

##### Distribution.

China (Zhejiang), Japan (Sakagami 2023).

##### Remarks.

This species is reported from China for the first time.

#### Sorolopha bathysema

Taxon classificationAnimaliaLepidopteraTortricidae

Diakonoff, 1973

3A800DA6-A7CE-563A-81C5-D8627F621985

Sorolopha bathysema Diakonoff, 1973: 95. TL: New Guinea. TD: NCB.

##### Specimens examined.

China • 1 ♂; Yunnan Prov., Xishuangbanna, Yexianggu; 22°10'N, 100°52'E; alt. 760 m; 8 Aug.2016; J.E. Li, T. Wang, and K.J. Teng leg.

##### Distribution.

China (Yunnan), New Guinea (Diakonoff 1973), Thailand (Kawabe 1989; Promwong and Pinkaew 2011).

##### Remarks.

This species is reported from China for the first time.

#### Sorolopha brunnorbis

Taxon classificationAnimaliaLepidopteraTortricidae

Razowski, 2009

C833F121-29D7-59CF-9D63-8A00BCA65F90

Sorolopha brunnorbis Razowski, 2009: 117. TL: Vietnam. TD: MNHU.

##### Distribution.

China (Guangdong, Guangxi, Hunan) (Zhao et al. 2017; Li et al. 2023), Vietnam (Razowski 2009).

##### Remarks.

Specimens from China were collected at elevations of 520–2,500 m.

#### Sorolopha bryana

Taxon classificationAnimaliaLepidopteraTortricidae

(Felder & Rogenhofer, 1874)

8E53A62E-5B78-549A-AC4D-D4F6DBF359EC

Penthina bryana Felder & Rogenhofer, 1874: pl. 137. TL: Sri Lanka. TD: NHMUK.
Sorolopha bryana : Diakonoff, 1973: 89.

##### Distribution.

China (Chongqing, Fujian, Guangdong, Guangxi, Guizhou, Taiwan) (Kawabe et al. 1992; Yu and Li 2009; Li et al. 2023; Sun and Li 2024), Sri Lanka (Felder and Rogenhofer 1874; Diakonoff 1973, 1982).

##### Remarks.

Specimens from China were collected at elevations of 300–2,200 m.

#### Sorolopha camarotis

Taxon classificationAnimaliaLepidopteraTortricidae

(Meyrick, 1936)

18E7DB56-D46D-5600-9BE9-64DFF0C4BC83

Argyroploce camarotis Meyrick, 1936: 612. TL: India. TD: NHMUK.
Olethreutes camarotis : Clarke, 1958: 491.
Eudemis camarotis : Diakonoff, 1968: 52.
Sorolopha camarotis : Diakonoff, 1973: 96.
Sorolopha longurus Liu & Bai, 1982: 168.
Sorolopha micheliacola Liu, 2001: 45.

##### Host plants.

Magnoliaceae: *Michelia sampaca*, *M. fuscata*; Myrtaceae: *Syzygium cumini* (Meyrick 1936; Diakonoff 1973; Liu and Li 2002; Robinson et al. 2023).

##### Distribution.

China (Fujian, Hainan, Jiangxi, Taiwan, Yunnan) (Liu and Bai 1982; Liu 2001; Liu and Li 2002; Yu and Li 2009; Sun and Li 2024), India (Meyrick 1936; Pathania et al. 2020; Naik and Shashank 2024), Indonesia (Diakonoff 1973), Malaysia (Robinson et al. 1994), Thailand (Kawabe 1989).

##### Remarks.

Specimens from China were collected at elevations of 550–2,300 m.

#### Sorolopha cyclotoma

Taxon classificationAnimaliaLepidopteraTortricidae

Lower, 1901

3885D3A6-9463-5DE4-BA9D-21B3493B5177

Sorolopha cyclotoma Lower, 1901: 74. TL: Australia. TD: SAMA.

##### Host plants.

Lauraceae: *Cinnamomum verum*, *Litsea glutinosa* (Robinson et al. 2023).

##### Distribution.

China (Hainan) (Sun and Li 2024), Australia (Lower 1901), New Guinea (Diakonoff 1973), Thailand (Kawabe 1989; Promwong and Pinkaew 2011), Vietnam (Le et al. 2013), Laos (Pinkaew and Phewphanh 2017).

##### Remarks.

Specimens from China were collected at elevations of 650–770 m.

#### Sorolopha dactyloidea

Taxon classificationAnimaliaLepidopteraTortricidae

Yu & Li, 2009

883FFF01-11BD-5310-B0C1-7CA5C2C861FD

Sorolopha dactyloidea Yu & Li, 2009: 5. TL: China. TD: NKU.

##### Distribution.

China (Yunnan) (Yu and Li 2009), Thailand (Muadsub and Pinkaew 2023).

##### Remarks.

Specimens from China were collected at an elevation of 650 m.

#### Sorolopha elaeodes

Taxon classificationAnimaliaLepidopteraTortricidae

(Lower, 1908)

ECA1EF48-B68F-53BB-A141-1186B5A6D7D1

Acanthothyspoda elaeodes Lower, 1908: 320. TL: Australia. TD: SAMA.
Argyroploce elaeodes : Meyrick, 1911: 281.
Argyroploce temenopis Meyrick, 1936: 614.
Olethreutes temenopis : Clarke, 1958: 535.
Eudemis temenopis : Diakonoff, 1968: 52.
Sorolopha elaeodes : Diakonoff, 1973: 62.

##### Food plant.

Lauraceae: *Persea americana* (Meyrick, 1936).

##### Distribution.

China (Hainan, Taiwan) (Meyrick 1936; Kawabe et al 1992; Sun and Li 2024), Australia (Lower 1908), New Guinea, Indonesia (Diakonoff 1973).

##### Remarks.

Specimens from China were collected at an elevation of 540 m.

#### Sorolopha euochropa

Taxon classificationAnimaliaLepidopteraTortricidae

Diakonoff, 1973

EDB02894-7064-5E7B-BE58-4BD3772FE35F

Sorolopha euochropa Diakonoff, 1973: 66. TL: Sumatra. TD: NCB.

##### Distribution.

China (Guangdong, Hainan) (Li et al. 2023), Indonesia (Diakonoff 1973), Thailand (Muadsub and Pinkaew 2023).

##### Remarks.

Specimens from China were collected at elevations of 30–640 m.

#### Sorolopha ferruginosa

Taxon classificationAnimaliaLepidopteraTortricidae

Kawabe, 1989

ED14DF5C-DCAF-5C16-8EF5-52F7E7CBD7B9

Sorolopha ferruginosa Kawabe, 1989: 30. TL: Thailand. TD: OPU.

##### Distribution.

China (Fujian, Guangdong, Guangxi, Guizhou, Yunnan) (Yu and Li 2009; Li et al. 2023), Thailand (Kawabe 1989).

##### Remarks.

Specimens from China were collected at elevations of 590–2,150 m.

#### Sorolopha ghilarovi

Taxon classificationAnimaliaLepidopteraTortricidae

Kuznetsov, 1988

E02241C4-E06F-5D99-B091-F164D257A953

Sorolopha ghilarovi Kuznetsov, 1988: 178. TL: Vietnam. TD: ZISP.

##### Specimens examined.

China • 1 ♂; Guangdong Prov., Jingxi County, Bangliang; 22°56'N, 106°26'E; alt. 820 m; 31 Jul. 2010; J.H. Huang leg.

##### Distribution.

China (Guangxi), Vietnam (Kuznetsov 1988).

##### Remarks.

This species is reported from China for the first time.

#### Sorolopha herbifera

Taxon classificationAnimaliaLepidopteraTortricidae

(Meyrick, 1909)

D587B6EA-66FB-5A8F-B66D-3D5EBAECA574

Fig. 3

Argyroploce herbifera Meyrick, 1909: 603. TL: India. TD: NHMUK.
Olethreutes herbifera : Meyrick, 1930: 604.
Eudemis herbifera : Diakonoff, 1968: 51.
Sorolopha herbifera : Diakonoff, 1973: 72.

##### Host plants.

Lauraceae: *Camphora officinarum* (Fletcher 1932; Diakonoff 1973; Robinson et al. 2023).

##### Biology.

**Figure 3.**
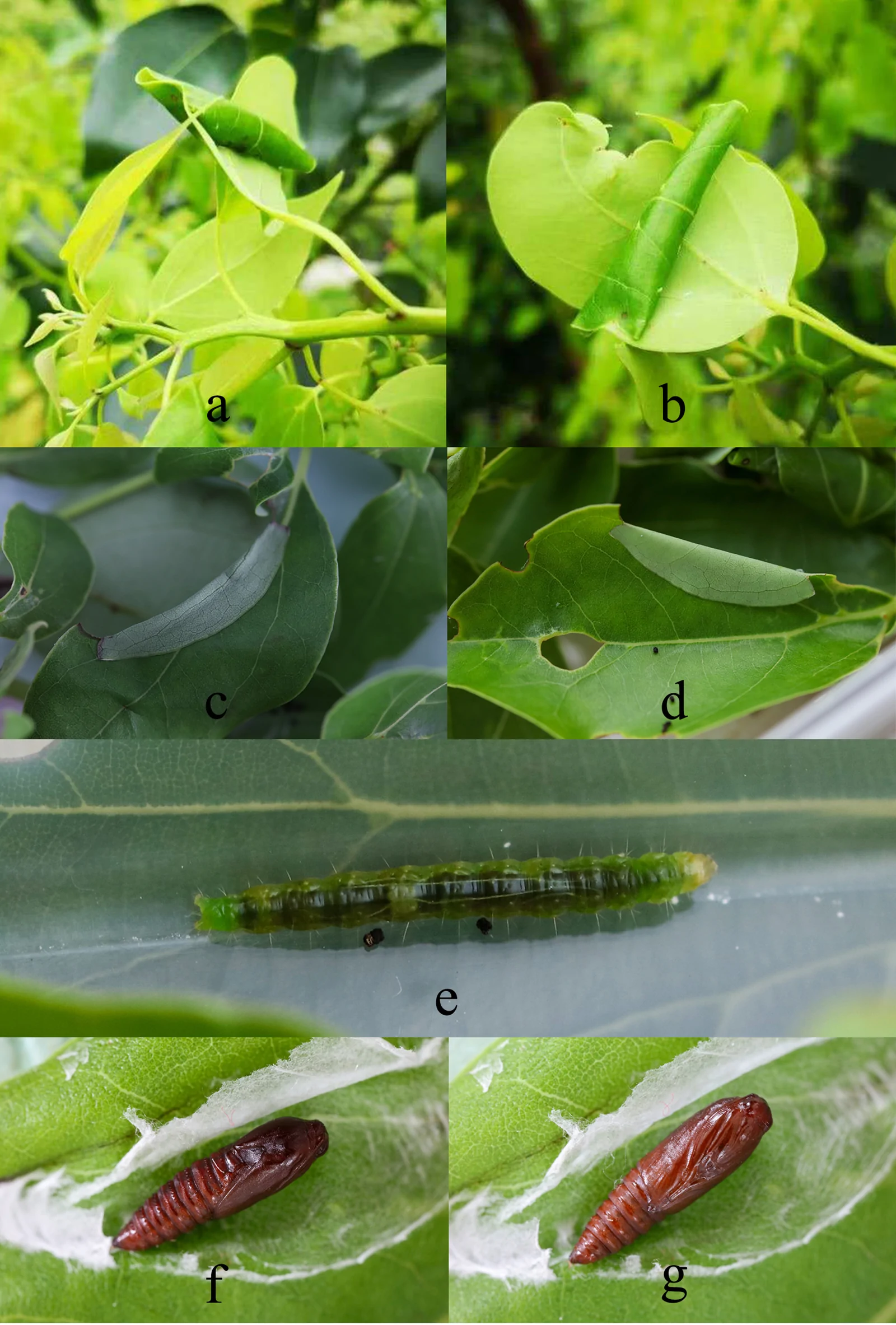
Larval stage of *Sorolopha herbifera* and their leaf shelters on *Camphora officinarum*. **a, b**. Leaf shelters on young leaves; **c, d**. Leaf shelters on mature leaves; **e**. Larva; **f, g**. Pupa.

In late May 2019, Prof. Shaobin Huang (Guangdong Eco-engineering Polytechnic, China) collected the larvae of this species on *Camphora officinarum* (L.) (Lauraceae) in Guangzhou. Observations revealed that the larvae construct solitary shelters by folding the leaf tip inward and securing it with silk, residing within this structure on tender leave (Fig. 3a, b). On mature leaves, larvae cut and fold back a portion of the leaf edge, secure it with silk, and pupate inside this modified shelter (Fig. 3c–g).

##### Distribution.

China (Fujian, Guangdong, Guangxi, Hainan, Taiwan, Yunnan) (Kawabe et al. 1992; Liu and Li 2002; Li et al. 2023; Sun and Li 2024), India (Meyrick 1909; Pathania et al. 2020; Naik and Shashank 2024), Indonesia (Diakonoff 1973), Malaysia, Nepal, Sri Lanka (Robinson et al. 1994; Koçak and Kemal 2012), Vietnam (Kuznetzov 1988; Le et al. 2013; Nedoshivina 2013), Thailand (Kawabe 1989; Pinkaew 2007).

##### Remarks.

Specimens from China were collected at elevations of 10–1,620 m.

#### Sorolopha identaeolochloca

Taxon classificationAnimaliaLepidopteraTortricidae

Yu & Li, 2009

BDA4E240-4562-5BE1-ADE8-0611FBE00573

Sorolopha identaeolochloca Yu & Li, 2009: 8. TL: China. TD: NKU.

##### Distribution.

China (Guangdong, Guizhou, Sichuan, Xizang, Yunnan) (Yu and Li 2009).

##### Remarks.

Specimens from China were collected at elevations of 590–2,300 m.

#### Sorolopha irirectangulata

Taxon classificationAnimaliaLepidopteraTortricidae

Hu & Yu sp. nov.

9561C324-5E57-5237-AEB9-0FE22DCA8E5A

https://zoobank.org/DFCFA2ED-3D07-4C9A-A60B-57516E6433D9

Figs 4, 6, 9

##### Type material.

***Holotype***: China • ♂; Yunnan Prov., Menghai, Damanlu village; 21°45'N, 100°9'E; alt. 1130 m; 1 Aug. 2019; K.J. Teng, C. Liu, and A.L. Wang leg.; genitalia slide no. YWX18319.

##### Diagnosis.

The male genitalia of *Sorolopha irirectangulata* sp. nov. are superficially similar to those of four congeners, *S. sphaerocopa* (Meyrick, 1930), *S. suthepensis* Patibhakyothin & Pinkaew, 2015, *S. asphaeropa* Diakonoff, 1973, and *S. asymmetrana* Kuznetsov, 1988, but can be readily distinguished by its symmetrical valvae, whereas the other four species possess asymmetrical valvae. Additionally, the valvae of *S. irirectangulata* sp. nov. have naked necks that are longer than those in *S. asphaeropa* and *S. asymmetrana*. The cucullus exhibits a rounded apex with a slight basal constriction; in contrast, the apex of cucullus is clavate with a strong basal constriction in *S. sphaerocopa* and slender in *S. suthepensis*.

##### Description.

**Figures 4, 5.**
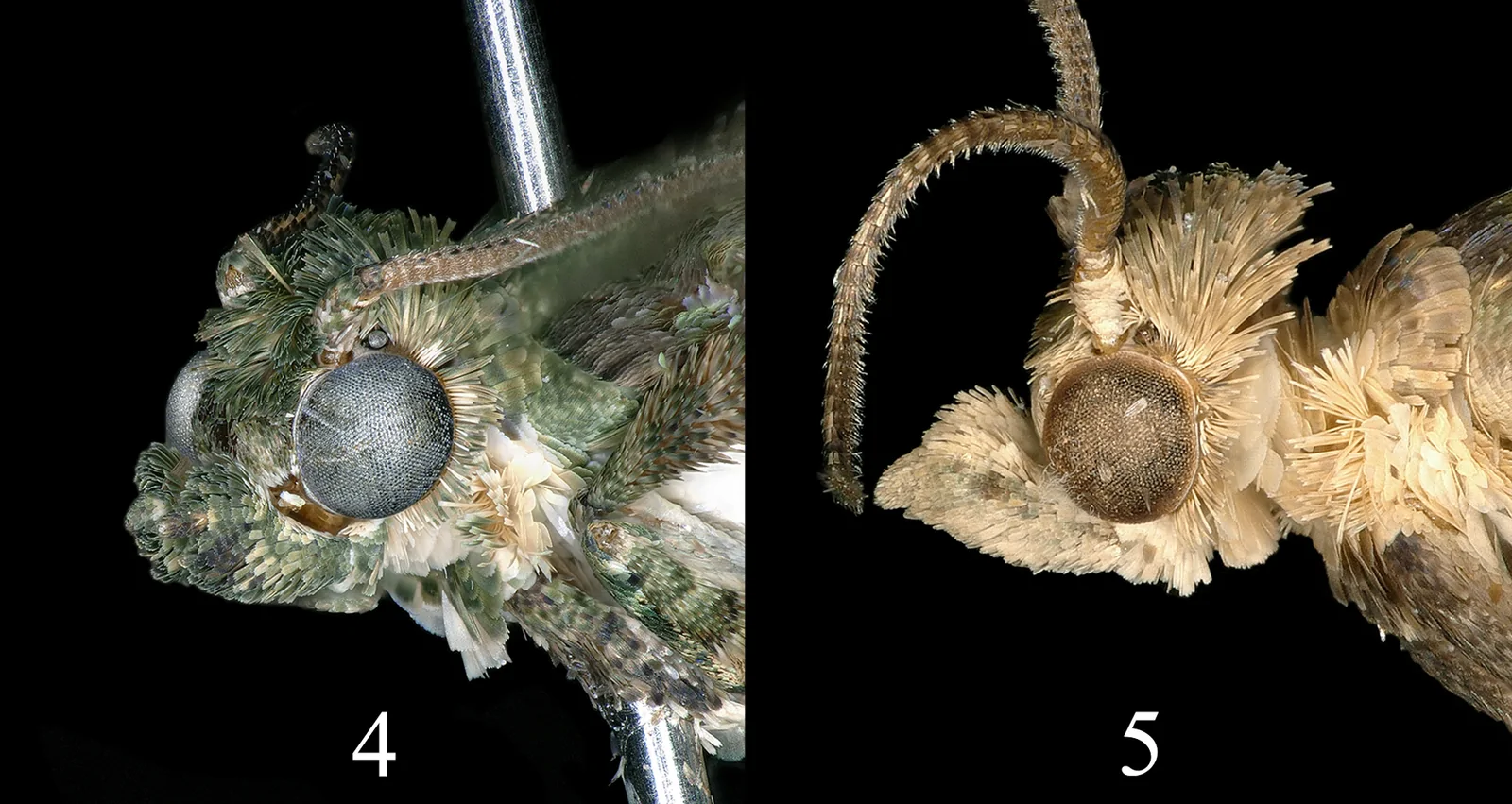
Heads of *Sorolopha* spp. **4**. *S. irirectangulata* sp. nov., holotype; **5**. *S. specialis* sp. nov., holotype.

**Figures 6–8.**
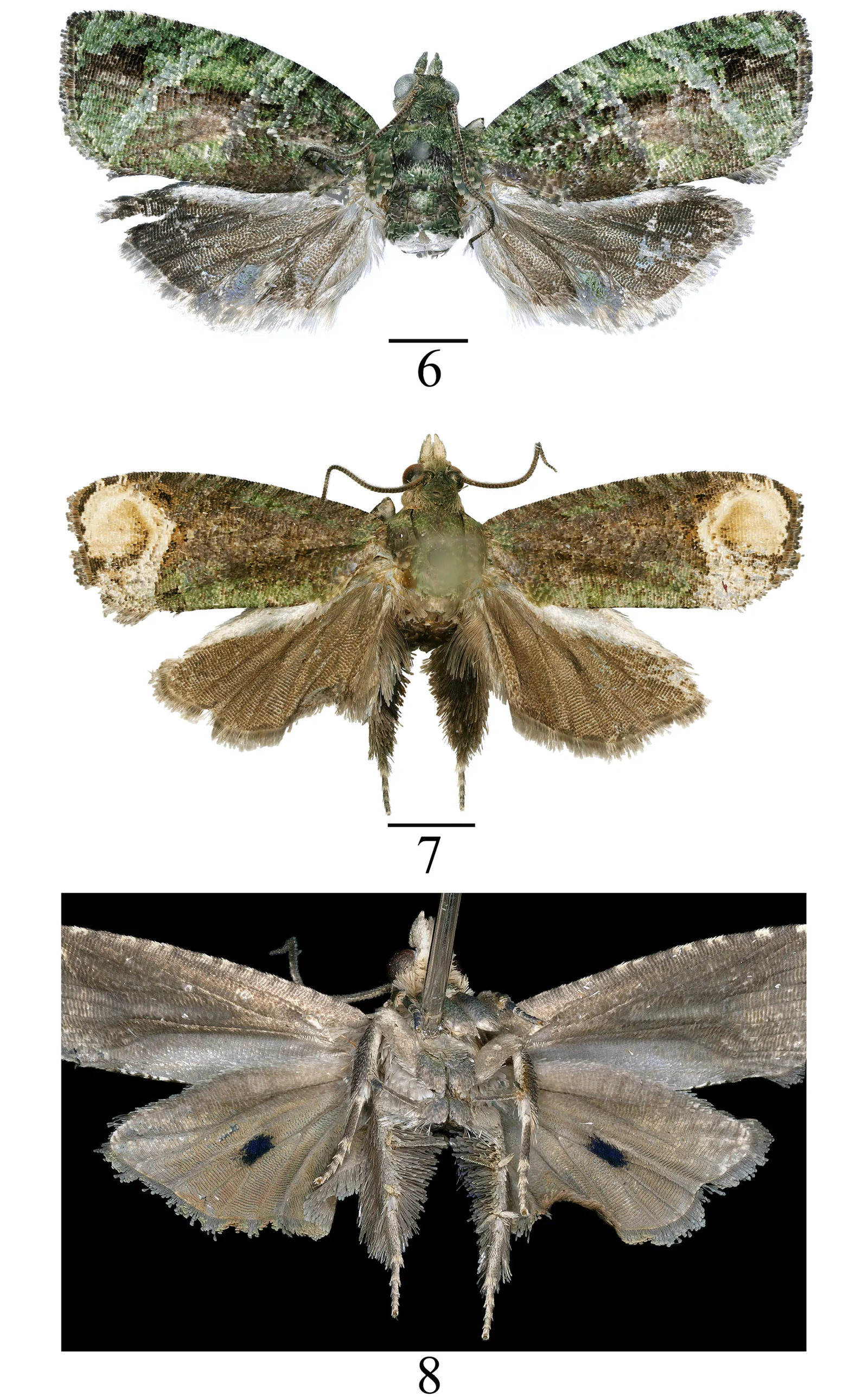
Adults of *Sorolopha* spp. **6**. *S. irirectangulata* sp. nov., male, holotype; **7**. *S. specialis* sp. nov., male, holotype; **8**. *S. specialis* sp. nov., holotype, sex scales on the underside of hindwing. Scale bars: 2.0 mm.

**Male** (Fig. 6). Forewing length 9.0 mm. ***Head*** (Fig. 4): Frons and vertex green. Antenna extending to middle of forewing, brown. Labial palpus ascending, green, dusted with brown and blackish fuscous; median segment slightly expanded, terminal segment relatively short and slightly sharp.

***Thorax***: Collar, tegula and dorsum green, dusted with brown, posterior crest distinct. Hind leg pale brown; tibia bright brown; tarsi blackish fuscous, with pale yellowish brown rings at apex of each subsegment. Forewing subrectangular, costa slightly arched in basal 1/2, nearly straight in distal 1/2, dorsum slightly oblique; upperside pale green, fasciae green suffused with deep brown; basal two pairs of costal strigulae green, distal seven pairs pale green; basal and subbasal faciae confluent, occupying basal 1/4 of wing, deep brown below 3A, with distal edge extending to middle of dorsum; media facia proximal edge extending from 2/5 of costa to mid-length of dorsum, distal edge running obliquely from 1/2 of costa to end of dorsum, upper part green with broken deep brown margins, deep brown on central area of cell, lower part brown suffused with pale green; postmedian fascia blackish brown, somewhat triangular, located between 2/5 length of R_4_ and distal 1/2 of M_3_, lower edge indistinct; cilia blackish brown; underside pale brown except for gray white costal strigulae, brown median fascia and white area overlapped by hindwing. Hindwing upperside brown, darkened towards apex, area overlapped by forewing white, with dense long scales along distal 1/2 of CuA_2_; anal region enlarged and thickened; cilia pale grey.

**Figures 9, 10.**
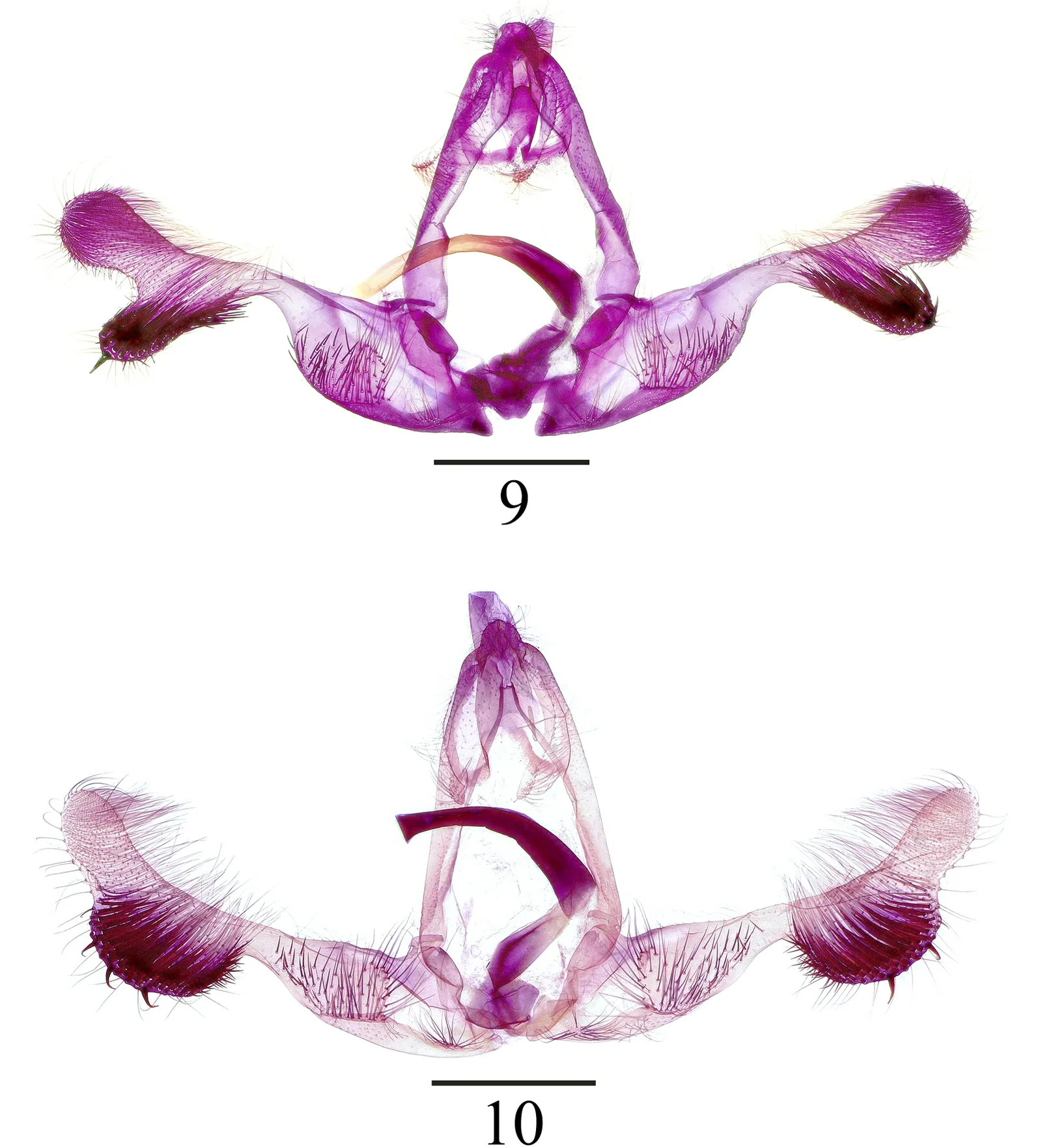
Male genitalia of *Sorolopha* spp. **9**. *S. irirectangulata* sp. nov., holotype, slide no. YWX18319; **10**. *S. specialis* sp. nov., holotype, slide no. YWX18110. Scale bars: 0.5 mm.

***Abdomen***: Terga 3–7 with tufts of long bristles on posterolateral margins in male. Male genitalia (Fig. 9): Tegumen high and narrow; uncus small and obtuse; socius slender, pendant, reaching midlength of tegumen, with sparse hairs, denser apically; gnathos narrow; valvae approximately symmetrical, slender, spindle-shaped basally, curved dorsally at apex; neck distinct, slightly curved, naked; sacculus with dense short spines; cucullus with ventral prominence elongate rounded, bearing long hairs basally and dense short spines distally, terminal part circular, densely hairy, slightly larger than ventral prominence, with a very short, broad stem; phallus curved, without cornuti.

**Female**. Unknown.

##### Genetics.

The partial mitochondrial COI sequence of the holotype has been deposited in GenBank under accession number PX852849. The average genetic distance between *S. irirectangulata* sp. nov. and other congeners was 9.48%, and the distance to its nearest neighbor was 7.16%; the nearest neighbor was *S. elaeodes* (Voucher numbers ANICB094-06 and ANICV1875-11). Because only the holotype was available for DNA extraction, intraspecific variation could not be assessed. Nevertheless, the observed interspecific divergence strongly supports the recognition of this new species as distinct.

##### Etymology.

The specific name is derived from the Latin prefix *ir*- (= not) and *rectangulatus* (= right-angled), referring to the shape of the sacculus in the male genitalia.

#### Sorolopha karsholti

Taxon classificationAnimaliaLepidopteraTortricidae

Kawabe, 1989

3E6D0BC0-C2A2-57BB-9C0B-164FD8ACF95A

Sorolopha karsholti Kawabe, 1989: 29. TL: Thailand. TD: OPU.

##### Distribution.

China (Yunnan) (Yu and Li 2009), Thailand (Kawabe 1989).

##### Remarks.

Specimens from China were collected at elevations of 2,140–2,300 m.

#### Sorolopha latiuscula

Taxon classificationAnimaliaLepidopteraTortricidae

Zhao, Bai & Yu, 2017

D4D0C5B9-79BD-5350-9F63-2C190A2B5946

Sorolopha nanlingica Zhao, Bai & Yu, 2017: 595. TL: China. TD: NKU.

##### Distribution.

China (Guangxi) (Zhao et al. 2017).

##### Remarks.

Specimens from China were collected at an elevation of 1,250 m.

#### Sorolopha liochlora

Taxon classificationAnimaliaLepidopteraTortricidae

(Meyrick, 1914)

55F81F41-243C-53C1-80FE-9EB08CECC80C

Argyroploce liochlora Meyrick, 1914: 771. TL: India. TD: NHMUK.
Olethreutes liochlora : Clarke, 1958: 559.
Sorolopha liochlora : Diakonoff, 1973: 64.

##### Distribution.

China (Hainan, Taiwan) (Yu and Li 2009; Heppner 2012; Sun and Li 2024), India (Meyrick 1914; Pathania et al. 2020; Naik and Shashank 2024), Indonesia (Diakonoff 1973), Thailand (Muadsub and Pinkaew 2023).

##### Remarks.

Specimens from China were collected at elevations of 40–1,050 m.

#### Sorolopha muscida

Taxon classificationAnimaliaLepidopteraTortricidae

(Wileman & Stringer, 1929)

C2FB6639-E05A-5065-B00F-9D08FAEE0B08

Argyroploce muscida Wileman & Stringer, 1929: 67. TL: China. TD: NHMUK.
Sorolopha muscida : Kawabe et al., 1992: 106.

##### Distribution.

China (Taiwan) (Wileman and Stringer 1929; Kawabe et al. 1992).

##### Remarks.

Specimens from China were collected at elevations of 480–3,100 m.

#### Sorolopha nanlingica

Taxon classificationAnimaliaLepidopteraTortricidae

Zhao, Bai & Yu, 2017

1375C3EA-E1BD-561A-98C6-403BB6468793

Sorolopha nanlingica Zhao et al., 2017: 594. TL: China. TD: NKU.

##### Distribution.

China (Guangdong) (Zhao et al. 2017; Li et al. 2023).

##### Remarks.

Specimen from China was collected at an elevation of 650 m.

#### Sorolopha phyllochlora

Taxon classificationAnimaliaLepidopteraTortricidae

(Meyrick, 1905)

CA7EE4E3-5DBE-5F7F-9AE1-1DB1BE6F0495

Platypeplus phyllochlora Meyrick, 1905: 585. TL: Sri Lanka. TD: NHMUK.
Eucosma phyllochlora : Meyrick, 1907: 137.
Argyroploce phyllochlora : Meyrick, 1909: 592.
Argyroploce ptilosema Meyrick, 1916: 563.
Olethreutes phyllochlora : Clarke, 1958: 535.
Olethreutes ptilosema : Clarke, 1958: 535.
Euedmis phyllochlora : Diakonoff, 1968: 52.
Sorolopha phyllochlora : Diakonoff, 1973: 68.

##### Host plants.

Lauraceae: *Cinnamomum camphora*, *Persea americana* (Meyrick 1936; Robinson et al. 2023).

##### Distribution.

China (Guangdong, Guangxi, Hainan, Hong Kong, Taiwan, Yunnan) (Liu and Li 2002; Yu and Li 2009; Li et al. 2023; Sun and Li 2024), Sri Lanka (Meyrick 1905), India (Meyrick 1916; Pathania et al. 2020; Naik and Shashank 2024), Thailand (Muadsub and Pinkaew 2023).

##### Remarks.

Specimens from China were collected at elevations of 40–3,100 m.

#### Sorolopha plinthograpta

Taxon classificationAnimaliaLepidopteraTortricidae

(Meyrick, 1931)

15788AD7-33A2-5D5B-8833-88AE7A7A9AA9

Argyroploce plinthograpta Meyrick, 1931: 135. TL: China. TD: NHMUK.
Eudemis plinthograpta : Issiki, 1957: 69.
Olethreutes plinthograpta : Clarke, 1958: 539.
Sorolopha plinthograpta : Diakonoff, 1973: 84.

##### Host plants.

Fabaceae: Pueraria montana var. lobata; Lauraceae: *Actinodaphne acuminata*, *Cinnamomum camphora*, *C. daphnoides*, *C. yabunikkei*, *Litsea coreana*, *Persea thunbergia*; Elaeocarpaceae: *Elaeocarpus japonicus* (Kawabe 1982; Nasu 2011, 2013; Robinson et al. 2023).

##### Distribution.

China (Guangdong, Hainan, Jiangxi, Taiwan, Yunnan) (Meyrick 1931; Diakonoff 1973; Liu and Bai 1982; Liu and Li 2002; Yu and Li 2009; Li et al. 2023; Sun and Li 2024), Japan (Issiki 1971; Diakonoff 1973), Thailand (Kawabe 1989; Pinkaew 2007), Korea (Byun et al. 1998), Russia (Kuznetsov 2001), Vietnam (Le et al. 2013).

##### Remarks.

Specimens from China were collected at elevations of 10–1,620 m.

#### Sorolopha plumboviridis

Taxon classificationAnimaliaLepidopteraTortricidae

Diakonoff, 1973

160F887C-A1AC-57EA-95D8-30E03D68CA61

Sorolopha plumboviridis Diakonoff, 1973: 83. TL: Indonesia. TD: NHMUK.

##### Distribution.

China (Taiwan, Yunnan) (Liu and Bai 1985; Yu and Li 2009; Sun and Li 2024), Indonesia (Diakonoff 1973), Thailand (Kawabe 1989).

##### Remarks.

Specimens from China were collected at elevations of 260–1,450 m.

#### Sorolopha rubescens

Taxon classificationAnimaliaLepidopteraTortricidae

Diakonoff, 1973

3BB4750F-15AC-5091-A821-38D026A25BE0

Sorolopha rubescens Diakonoff, 1973: 70. TL: Indonesia. TD: NHMUK.
Sorolopha chlorotica Liu *& B*ai, 1985: 136.

##### Distribution.

China (Guangdong, Hainan, Taiwan, Yunnan) (Liu and Bai 1985; Kawabe et al. 1992; Yu and Li 2009; Li et al. 2023; Sun and Li 2024), Indonesia (Diakonoff 1973), Thailand (Kawabe 1989), Vietnam (Le et al. 2013).

##### Remarks.

Specimens from China were collected at elevations of 50–2,270 m.

#### Sorolopha saitoi

Taxon classificationAnimaliaLepidopteraTortricidae

Kawabe, 1989

BDAF3A86-AD23-5A4C-BEC2-FB7266CDB880

Sorolopha saitoi Kawabe, 1989: 31. TL: Thailand. TD: OMU.

##### Distribution.

China (Guangdong, Yunnan) (Li et al. 2023), Thailand (Kawabe 1989).

##### Remarks.

Specimens from China were collected at elevations of 50–1,450 m.

#### Sorolopha semiculta

Taxon classificationAnimaliaLepidopteraTortricidae

(Meyrick, 1909)

4CAA7D4D-6721-5929-B465-3200FBAED76D

Argyroploce semiculta Meyrick, 1909: 604. TL: Sri Lanka. TD: NHMUK.
Argyroploce heteraspis Meyrick, 1936: 614.
Olethreutes semiculta : Clarke, 1958: 547.
Sorolopha semiculta : Diakonoff, 1973: 76.

##### Host plants.

Lauraceae: *Alseodaphne semicarpifolia*, *Persea americana* (Meyrick 1916, 1936; Diakonoff 1973).

##### Distribution.

China (Chongqing, Guangxi, Hainan, Taiwan, Yunnan) (Meyrick 1936; Kawabe et al. 1992; Liu and Li 2002; Yu and Li 2009; Sun and Li 2024), Sri Lanka (Meyrick 1909), India, Soloman Is., New Guinea (Diakonoff 1973), Vietnam (Kuznetzov 1988; Le et al. 2013), Thailand (Pinkaew 2007).

##### Remarks.

Specimens from China were collected at elevations of 190–1,100 m.

#### Sorolopha singularis

Taxon classificationAnimaliaLepidopteraTortricidae

Zhao, Bai & Yu, 2017

E63694FA-919E-50DF-B62A-C1958D75C887

Sorolopha singularis Zhao et al., 2017: 597. TL: China. TD: NKU.

##### Distribution.

China (Guangxi, Hainan, Xizang, Yunnan) (Zhao et al. 2017).

##### Remarks.

Specimens from China were collected at elevations of 40–1,000 m.

#### Sorolopha specialis

Taxon classificationAnimaliaLepidopteraTortricidae

Zhu & Yu sp. nov.

653FA558-BEEF-5E63-B20C-F214DE95368E

https://zoobank.org/56EE3B6C-EBCD-418C-9AC5-AAB265182C9C

**Figures 11, 12.**
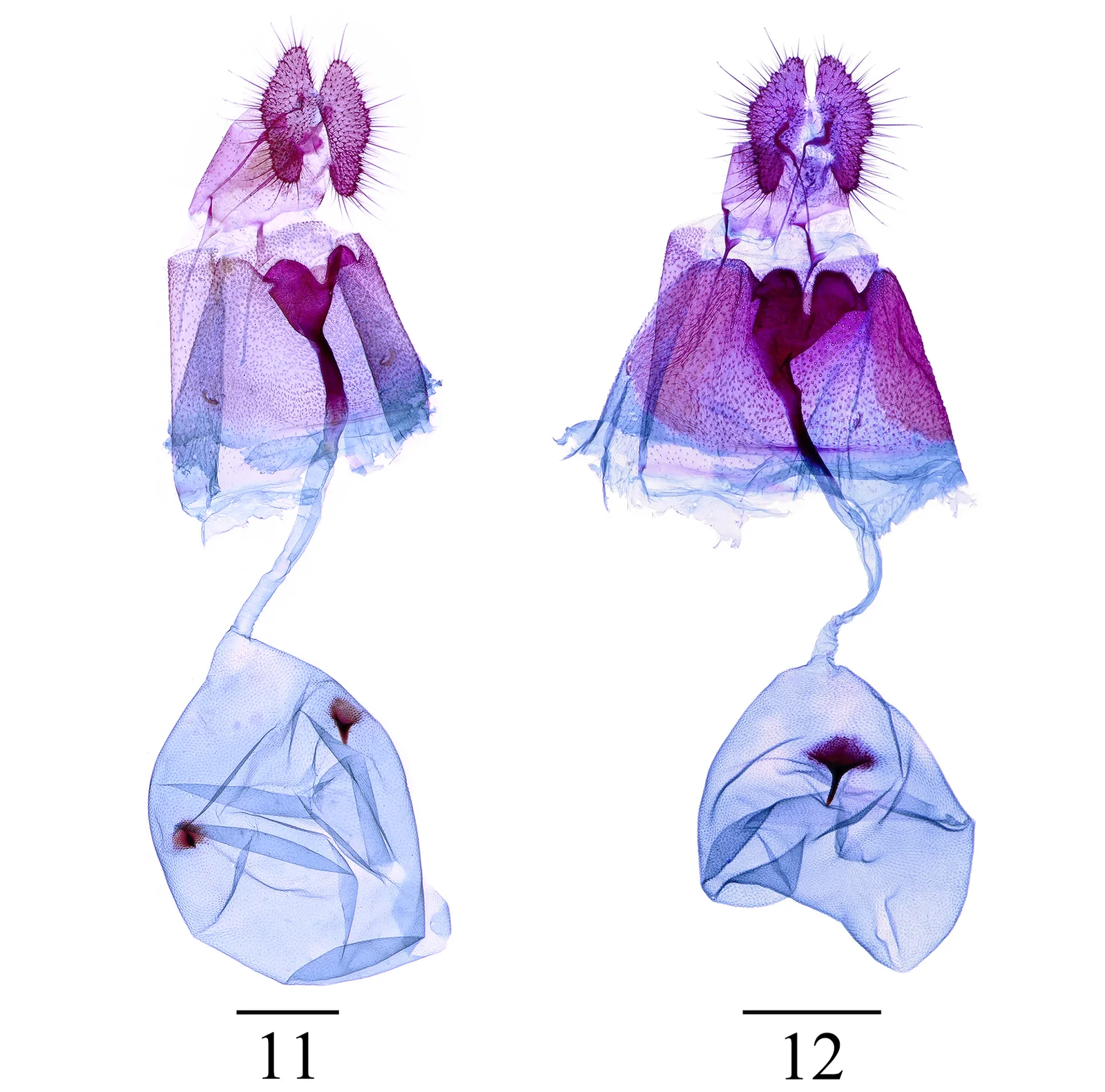
Female genitalia of *Sorolopha* spp. **11**. *S. specialis* sp. nov., paratype, slide no. YWX18111; **12**. *S. suthepensis*, slide no. YWX18112. Scale bars: 0.5 mm.

Figs 5, 7, 8, 10, 11

##### Type material.

***Holotype***: China • ♂; Guangxi Zhuang Autonomous Region, Pingxiang, Shaoping forest farm; 22°4'N, 106°55'E; alt. 280 m; 1 Apr. 2013; X.F. Yang leg.; genitalia slide no. YWX18110. ***Paratypes***: China • 2 ♂♂, 1 ♀; same collection data as for holotype; 2 Apr. 2013 • 1 ♂; Hainan Prov., Yinggeling, Hongkan; 19°48'N, 109°30'E; alt. 510 m; 17 May. 2015; P.X. Cong, W. Guan, and S. Hu leg. • 1 ♂; Hainan Prov., Wuzhishan Reserve; 18°54'N, 109°40'E; alt. 740 m; 13 Apr. 2009; Q. Jin and B.B. Hu leg.

##### Diagnosis.

*Sorolopha specialis* sp. nov. is very similar to *S. ferruginosa* and *S. nanlingica* in forewing pattern, but can be distinguished by the presence of blackish androconial scales on the lower surface of the hindwing in males. In male genitalia morphology, *S. specialis* sp. nov. resembles several congeners, including *S. ferruginosa*, *S. nanlingica*, *S. baribaris*, and *S. viridis* Sakagami, 2023. It differs from *S. ferruginosa* by the valva having a straight neck and a cucullus whose distal part is ~ 2/3 the length of the ventral prominence; in *S. ferruginosa*, the valva has a gently curved neck and a cucullus with distal part ~ 1/2 the length of the ventral prominence. *Sorolopha specialis* sp. nov. differs from *S. nanlingica* and *S. baribaris* in the male genitalia by the valva with a relatively long neck and a semi-rounded ventral prominence; in contrast, these two species have a valva with a short neck and a ventral prominence of the cucullus that gradually widens outward. *Sorolopha specialis* sp. nov. differs from *S. viridis* in that the distal part of the cucullus is uniform in width and ~ 2× as wide as the neck of the valva; whereas in *S. viridis*, the distal part of the cucullus gradually narrows outward and is approximately equal in width to the neck of the valva.

##### Description.

**Adult** (Fig. 5). Forewing length 6.0–7.5 mm. ***Head*** (Fig. 7): Frons creamy white. Vertex roughly scaled, pale brown suffused with green. Antenna extending to middle of forewing, brown. Labial palpus porrect, creamy white, suffused with pale grey and green laterally; median segment expanded, terminal segment relatively short, stout.

***Thorax***: Collar brown suffused with green basally and pale brown apically. Tegula green basally and brown distally. Dorsum smooth, green suffused with brown, posterior crest indistinct. Hind leg pale brown, tibia and basal subsegment of tarsus with dense, blackish scales in males. Forewing subrectangular; costa nearly straight except for a slightly curved apex, termen slightly oblique; upperside grayish brown; basal seven pairs of costal strigulae green, distal two pairs white; inner margin of wing and area between fold and dorsum green, with tiny, dark brown spots along dorsum; markings indistinguishable, indicated by a few fragmented, ferruginous spots edged with indistinct, blackish lines on central area of wing; apical 1/3 of wing (from R_3_ to tornus) with white ground color, bearing a narrow, crescent-shaped subapical mark extending from middle of R_4_ to distal 2/5 of CuA_1_ and from 1/3 length of M_1_ to CuA_1_ on termen, dark brown suffused with ferruginous, with inner side surrounded by brown, curving lines, and scattered, pale brown scales between crescent mark and apex; cilia pale brown, with a brown baseline; underside pale brown, except for grayish-white costal strigulae and a white area overlapped by hindwing. Hindwing upperside brown except for white area overlapped by forewing; cilia pale brown, with a brown baseline; underside pale brown, with an elongate-ovoid patch of dense, blackish androconial scales on lower angle of cell (Fig. 8).

***Abdomen***: Terga 3–7 with tufts of long bristles on posterolateral margins in males. Male genitalia (Fig. 10): Tegumen narrow, subtriangular; uncus small and obtuse; socius slender, pendant, reaching mid-length of tegumen, with short spines at apex; gnathos weak, a narrow band fused with ventral surface of subscaphium; valvae symmetrical, moderate in width, narrow at middle; sacculus with ventral margin evenly curved, with sparse short spines; neck short, ~ 1/3× maximum width of cucullus, naked; cucullus with basal 3/5 expanded to a broad, rounded ventral prominence, densely setose along ventral edge, distal 2/5 narrow, apex rounded; phallus slender, curved, moderate in length, without cornuti. Female genitalia (Fig. 11): Papillae anales narrow, densely setose; apophyses anterior equal to posterior apophysis in length; sterigma aciculate, funnel-shaped, inverted triangular, with caudal margin protruding and deeply concave medially; ductus bursae membranous, approximately as long as corpus bursae; colliculum 1/3× length ductus bursae, inception of ductus seminalis posterior to anterior 1/4 length; corpus bursae large, ovoid, weakly granulated; two signa roughly equal in size, granulated, short horn-shaped.

##### Etymology.

The specific name is derived from the Latin *specialis* (= unique), referring to the blackish androconial scales on the hindwing of the male.

#### Sorolopha sphaerocopa

Taxon classificationAnimaliaLepidopteraTortricidae

(Meyrick, 1929)

9615D267-D489-593F-8DF4-7C32CEE58684

Argyroploce sphaerocopa Meyrick, 1929: 719. TL: Vietnam. TD: MNHN.
Choganhia sphaerocopa : Razowski, 1960: 387.
Acanthothyspoda sphaerocopa : Diakonoff, 1966: 56.
Eudemis sphaerocopa : Diakonoff, 1968: 52.
Sorolopha sphaerocopa : Diakonoff, 1973: 86.

##### Host plants.

Lauraceae: *Cinnamomum* sp., *C. camphora*, *C. yabunikkei*, *Machilus thunbergii*, *M. japonica*; Elaeocarpaceae: *Elaeocarpus japonicus* (Diakonoff 1973; Kawabe 1982; Nasu 2013).

##### Distribution.

China (Taiwan) (Kawabe et al. 1992; Sun and Li 2024), Vietnam (Meyrick 1929; Kuznetzov 1988; Le et al. 2013), India, Indonesia (Diakonoff 1973), Japan (Kawabe 1982), Thailand (Kawabe 1989), Russia (Kuznetsov 2001).

##### Remarks.

Specimens from China were collected at elevations of 100–1200 m.

#### Sorolopha stygiaula

Taxon classificationAnimaliaLepidopteraTortricidae

(Meyrick, 1933)

74D0253C-E5BC-56D9-B41E-B1F020DEF2C7

Argyroploce stygiaula Meyrick, 1933: 419. TL: Indonesia. TD: NHMUK.
Olethreutes stygiaula : Clarke, 1958: 552.
Eudemis stygiaula : Diakonoff, 1968: 52.
Sorolopha stygiaula : Diakonoff, 1973: 97.

##### Host plants.

Pandanaceae: *Pandanus* spp. (Meyrick 1933; Diakonoff 1973).

##### Distribution.

China (Guangdong, Jiangxi, Taiwan, Yunnan) (Yu and Li 2009; Li et al. 2023; Sun and Li 2024), Indonesia, India (Meyrick 1933; Diakonoff 1968), Thailand (Pinkaew 2007).

##### Remarks.

Specimens from China were collected at elevations of 570–2,150 m.

#### Sorolopha suthepensis

Taxon classificationAnimaliaLepidopteraTortricidae

Patibhakyothin & Pinkaew, 2015

353CBC97-0CEF-5BEE-9B9A-C07A742F712A

Sorolopha suthepensis Patibhakyothin & Pinkaew, 2015: 136. TL: Thailand. TD: KKIC.

##### Specimens examined.

China • 1 ♂; Guangxi Zhuang Autonomous Region, Longzhou County, Nonggang; 22°27'N, 106°57'E; alt. 280 m; 24 Jul. 2012; X.F. Yang leg. • 1 ♂; Xizang Zang Autonomous Region, Moto County, Beibeng; 29°14'N, 95°10'E; alt. 990 m; 12 Aug. 2017; M.J. Qi and X.F. Yang leg. • 2 ♂♂; Yunnan Prov., Baoshan County, Baihualing; 25°17'N, 98°48'E, alt. 1,470 m; 28–30 Jul. 2013; S.R. Liu, Y.Q. Wang, and K.J. Teng leg. • 1 ♂, 6 ♀♀; same collection data as for preceding; 25°16'N, 98°45'E; alt. 1,580 m; 4–6 Aug.2015; J.X. Zhao and H. Wei leg. • 4 ♂♂, 5 ♀♀; Yunnan Prov., Tengchong County, Linjiapu; 25°17'N, 98°42'E; alt. 2,150 m; 15 Aug. 2014; S.R. Liu, H. Rong, and K.J. Teng leg. • 1 ♂, 1 ♀; Yunnan Prov., Longling County, Xiaoheishan reserve; 24°31'N, 98°50'E; alt. 1,970 m; 18 Jul. 2013; S.R. Liu, Y.Q. Wang, K.J. Teng leg.

##### Description.

**Female**. Forewing length 15.5–16.0 mm, externally similar to the male. Genitalia (Fig. 12): papillae anales narrow; apophyses posteriores equal to apophyses anteriores in length; sterigma funnel-shaped and aciculate, with an expanded caudal margin that bears a deep median incision; colliculum sclerotized and elongate, ~ 1/3× length ductus bursae; ductus bursae slightly longer than corpus bursae, with ductus seminalis originating from its midregion; corpus bursae oval; signum single, large and horn-shaped, with an enlarged base.

##### Distribution.

China (Guangxi, Xizang, Yunnan), Thailand (Patibhakyothin et al. 2015).

##### Remarks.

This species is newly recorded from China, and the female is described for the first time.

#### Sorolopha tenuirurus

Taxon classificationAnimaliaLepidopteraTortricidae

Liu & Bai, 1982

AFBD7967-A957-5D04-A9F7-54BAB5C70022

Sorolopha tenuirurus Liu & Bai, 1982: 168. TL: China. TD: IZAS.

##### Distribution.

China (Sichuan, Xizang, Yunnan) (Liu and Bai 1982; Liu and Li 2002; Yu and Li 2009).

##### Remarks.

Specimens from China were collected at elevations of 1,450–2,300 m.

## Discussion

Adults of *Sorolopha* can be readily distinguished from other genera within the tribe Olethreutini by morphological traits, especially those of the genitalia. However, closely related species within *Sorolopha* are often difficult to differentiate because of their similar external appearance or genital structures. DNA barcodes could serve as a valuable tool for accurately identifying most *Sorolopha* species. In the present study, however, the genetic distances among 31 species-level clades of *Sorolopha* exhibit a continuous spectrum of variation: 2.89–3.23% between *S. elaeodes* and two specimens of the Vietnamese *Sorolopha* sp. (vouchers ASTOR370-11 and ASTOR371-11); 3.57% between *S. herbifera* and the Australian *Sorolopha* sp. A (GenBank accession numbers KF403038, KF397720, KF400433); 3.52–4.21% between *S. semiculta* and *Sorolopha* sp. AAL8452 from Papua New Guinea; 4.01% between the two Vietnamese sequences of *S. arctosceles* (vouchers ASTOR299-11 and ASTOR376-11); and 5.06–5.42% between the two Vietnamese *Sorolopha* sp. clades; distances extended up to 16.61% in the most divergent comparisons.

This continuous gradation of interspecific genetic divergence complicates DNA barcode-based species delimitation in *Sorolopha*. The maximum intraspecific distance within the genus was 2.38% for *S. cyclotoma*, which overlaps substantially with the minimum interspecific distance (2.89%) and leaves no distinct barcode gap. By contrast, such continuous genetic variation has not been observed in other Olethreutini genera examined previously, such as *Hiroshiinoueana* Kawabe, 1978, *Sycacantha* Diakonoff, 1959, *Syntozyga* Lower, 1901, and *Statherotmantis* Diakonoff, 1973 (Fei et al. 2018; Feng et al. 2019; Yang et al. 2021; Jing et al. 2024), most species of which exhibit interspecific divergences greater than 4%. Notably, intraspecific genetic divergences in the other 14 *Sorolopha* species-level clades were all below 0.93%. For *S. cyclotoma*, the genetic distance was 0.62% between two Vietnamese specimens and 0.15–0.46% among three Australian specimens. These results suggest that a threshold of less than 1% can be reliably applied to intraspecific identification in *Sorolopha*. Furthermore, despite its relatively high intraspecific divergence, *S. cyclotoma* was separated from all congeners by distances exceeding 6.25%. Similarly, species pairs with low interspecific distances generally showed low intraspecific differentiation: *S. elaeodes* had an intraspecific divergence of no more than 0.15%; the two sequences of the Vietnamese *Sorolopha* sp. (vouchers ASTOR370-11 and ASTOR371-11) differed by 0.77%; *S. herbifera* and Australian *Sorolopha* sp. A both showed no intraspecific divergence; and *S. semiculta* had an intraspecific distance of 0.46%.

Overall, there is no universal and clear-cut genetic threshold for DNA barcode-based species delimitation in *Sorolopha*. Nevertheless, each species appears to possess a distinct genetic gap between its intraspecific variation and its interspecific divergence from congeners. Therefore, particular caution is warranted when divergence between *Sorolopha* samples reaches 2%, and species identification should be integrated with morphological evidence. The barcode data for *Sorolopha* effectively support the applicability of the conventional 2% DNA barcode threshold for lepidopteran species, a pattern that differs from the aforementioned Olethreutini genera, in which the minimum interspecific divergence is markedly higher than 2%.

In addition, the genus *Sorolopha* may harbour cryptic species. For instance, among sequences deposited by the same research institution in Vietnam, specimens of the unidentified *Sorolopha* sp. and *S. arctosceles* each clustered into two deeply divergent clades; similarly, *S. archimedias* specimens from three different regions were split into two clades. In each case, the genetic divergence between the respective pairs of clades far exceeds the typical threshold used to delimit species.

The continuous genetic divergence in *Sorolopha* may result from the broad geographical sampling range of the present study, which may increase genetic variation among populations. At the same time, species misidentification cannot be excluded. Publicly available sequences originate from diverse independent studies and inevitably contain occasional misidentifications. For example, the newly generated sequences of *S. bryana* and *S. suthepensis* obtained in this study clustered with *S. sphaerocopa* (Voucher ASTOR512-11) and with four Vietnamese sequences identified as *S. arctosceles* (vouchers ASTOR372-11, ASTOR373-11, ASTOR374-11, and ASTOR375-11), respectively, showing genetic distances of 0% and 0.5%. Morphologically, *S. sphaerocopa* closely resembles *S. bryana*, and *S. arctosceles* closely resembles *S. suthepensis* in external appearance; however, they are clearly distinguishable based on genitalia characteristics. In this study, species identifications were based on both external morphology and detailed examination of genitalia. We therefore hypothesize that the original identifications of the aforementioned *S. sphaerocopa* and *S. arctosceles* specimens may have relied solely on external morphology, resulting in erroneous species assignment.

Based on morphology of the male genitalia, particularly the shape of the valva and socii, Diakonoff (1973) divided *Sorolopha* into six species groups: the *cyclotoma* group (included here: *S. archimedias*, *S. cyclotoma*, *S. elaeodes*, *S. irirectangulata* sp. nov., *S. ferruginosa*, and *S. baribaris*), the *liochlora* group (included here: *S. brunnorbis* and *S. arctosceles*), the *herbifera* group (included here: *S. herbifera*, *S. semiculta*, and *S. plinthograpta*), the *sphaerocopa* group (included here: *S. delochlora*, *S. suthepensis*, *S. karsholti*, *S. asymmetrana*, and *S. atmochlora*), the *bryana* group (included here: *S. bryana*, *S. tenuirurus*, and *S. ghilarovi*), and the *stygiaula* group (included here: *S. stygiaula*, *S. agana*, *S. camarotis*, and *S. singularis*). However, our maximum-likelihood phylogenetic analysis based on COI sequences did not support this classification, and the branching pattern among the defined groups is complex. The *cyclotoma* group is split into four distantly related clades. Only two species of the *liochlora* group were sampled here; nevertheless, *S. arctosceles* specimens may represent three distinct species-level units that do not form an independent assemblage. The status of the *herbifera* group remains ambiguous: its three species included here, together with two unidentified species from Australia and Papua New Guinea, form a single independent clade. Members of the *sphaerocopa* group are nested within one large clade that also includes five species from the *cyclotoma* and *bryana* groups plus two unidentified species. The *bryana* group falls into two well-separated clades, whereas the *stygiaula* group is paraphyletic. In addition, the present analysis also includes six unidentified species, further hindering rigorous testing of Diakonoff’s traditional classification. Because single-gene phylogenies cannot replace comprehensive multi-locus phylogenetic analyses, further morphological reassessment and expanded molecular sampling are necessary to resolve relationships within *Sorolopha*.

Males of Olethreutinae generally possess diverse modified androconial scales for scent production and dissemination, though functional evidence remains limited for most species. Typical structures include costal fold scales on the forewing, abdominal or wing scent patches, and specialized hair pencils on the hind tibiae (Diakonoff 1973; Razowski 1989; Horak 2006; Gilligan et al. 2008). In *Sorolopha*, males often bear prominent tufts of sex scales on the hind legs, on the sides of the abdomen, and in the anal area of the hindwing. The new species described here, *S. specialis* sp. nov., is remarkable for possessing a distinct patch of blackish androconial scales on the underside of the center of the hindwing—a feature not previously recorded in the genus. Among Olethreutini species known from China, this character has been observed in only two species: *Statherotis discana* (Felder & Rogenhofer, 1875) and *Cephalophyes porphyrea* Diakonoff, 1973. However, in these two species, the blackish androconial scales occur on the underside of the forewings and in the centre of the hindwings in males.

In China, *Sorolopha* species are more commonly found in Guangdong, Hainan, and Taiwan provinces, which may be associated with the widespread distribution of plants of the family Lauraceae in these regions. To date, host plants have been documented for only 13 species in this genus, eight of which include Lauraceae among their hosts.

## Supplementary Material

XML Treatment for Sorolopha

XML Treatment for Sorolopha aeolochlora

XML Treatment for Sorolopha agana

XML Treatment for Sorolopha angustorbis

XML Treatment for Sorolopha archimedias

XML Treatment for Sorolopha asphaeropa

XML Treatment for Sorolopha baribaris

XML Treatment for Sorolopha bathysema

XML Treatment for Sorolopha brunnorbis

XML Treatment for Sorolopha bryana

XML Treatment for Sorolopha camarotis

XML Treatment for Sorolopha cyclotoma

XML Treatment for Sorolopha dactyloidea

XML Treatment for Sorolopha elaeodes

XML Treatment for Sorolopha euochropa

XML Treatment for Sorolopha ferruginosa

XML Treatment for Sorolopha ghilarovi

XML Treatment for Sorolopha herbifera

XML Treatment for Sorolopha identaeolochloca

XML Treatment for Sorolopha irirectangulata

XML Treatment for Sorolopha karsholti

XML Treatment for Sorolopha latiuscula

XML Treatment for Sorolopha liochlora

XML Treatment for Sorolopha muscida

XML Treatment for Sorolopha nanlingica

XML Treatment for Sorolopha phyllochlora

XML Treatment for Sorolopha plinthograpta

XML Treatment for Sorolopha plumboviridis

XML Treatment for Sorolopha rubescens

XML Treatment for Sorolopha saitoi

XML Treatment for Sorolopha semiculta

XML Treatment for Sorolopha singularis

XML Treatment for Sorolopha specialis

XML Treatment for Sorolopha sphaerocopa

XML Treatment for Sorolopha stygiaula

XML Treatment for Sorolopha suthepensis

XML Treatment for Sorolopha tenuirurus

## Additional information

### Artificial Intelligence (AI) use

The authors accept full responsibility for the content of the manuscript, including the disclosure of any use of AI.

No AI tools were used in the preparation of this manuscript.

### Author ORCIDs

Z. Hu https://orcid.org/0009-0006-4335-5030

X. Chang https://orcid.org/0009-0008-9330-309X

Y. Ma https://orcid.org/0009-0007-9226-7878

E. Li https://orcid.org/0009-0009-1965-6652

C. Guo https://orcid.org/0009-0000-6807-192X

H. Yu https://orcid.org/0000-0003-1249-5461

### Data availability

All of the data that support the findings of this study are available in the main text or Supplementary Information.

## Supplementary materials

Supplementary material 1 Species sampled for the molecular analysis (the newly generated sequences in this study are indicated by an asterisk) Data type xlsx https://binary.pensoft.net/file/1814263This dataset is made available under the Open Database License (http://opendatacommons.org/licenses/odbl/1.0/). The Open Database License (ODbL) is a license agreement intended to allow users to freely share, modify, and use this Dataset while maintaining this same freedom for others, provided that the original source and author(s) are credited. Zhurong Hu, Xiuping Chang, Yuxuan Ma, Ershuai Li, Changyi Guo, Haili Yu

Supplementary material 2 Pairwise distances calculated within and between *Sorolopha* species resulting from COI gene dataset Data type xlsx https://binary.pensoft.net/file/1814264This dataset is made available under the Open Database License (http://opendatacommons.org/licenses/odbl/1.0/). The Open Database License (ODbL) is a license agreement intended to allow users to freely share, modify, and use this Dataset while maintaining this same freedom for others, provided that the original source and author(s) are credited. Zhurong Hu, Xiuping Chang, Yuxuan Ma, Ershuai Li, Changyi Guo, Haili Yu
